# On Some Aspects of Memory

**Published:** 1901-01-12

**Authors:** John Aikman


### ON SOME ASPECTS OF MEMORY.

By JOHN AIKMAN, M.D.

### I.—PERSONAL AND RELATIVE.

THE first and the last glimpse of our memory record is hidden. The breath which gave us independent life, the food which gave imprint to our earlier impression, are both forgotten. Our last spoken word, and the sigh with which we relinquish our individuality, are beyond the pale of our consciousness. Yet our first act of breathing is repeated to the end of our lives, in direct sequence. Our last word is cherished when we ourselves have passed beyond all activity.

Our first record is useful to ourselves. Our later records are shared by, and in some measure influence, the lives of others.

In all nature there is memory. The blade of grass which we crush under our feet revives and recovers. But our act is stamped upon its growth and reacts upon its root. Even inanimate nature has its records. The stratified rock tells us of periodic deposit, and preserves for us, not only the fossil, but a cast of the form and proportion of what then existed. The primary rock, barren of life form, tells us of the upheavals of nature, of the influence of heat, and of all that made activity before life was.

Memory is the record of activity: the greater the activity, the stronger is the memory. It is, or should be, something which makes, or mars, the future.

When we forget this principle we limit the measure of memory. We tend to make it an individual possession, which it is; but it is far more. We overlook the extent to which it dominates our habits, and the records which they make upon the lives of others and the world at large. These outer records we can never efface. They pass beyond our consciousness and control. But in the lives of others they are like the fossil in the rock, which not only keeps its place, but makes an indent which only final dissolution can efface.

Of recent memory we are conscious, and we share it with our friends of free will, if not always with judgment. Unconscious memory is our own in so far as it is the mainspring of habit, and saves us trouble. It belongs to our friends and to the world at large, in all that they gather for good or for evil from our thoughtlessness.

Our pet animals teach us lessons. With them memory passes into convenient habits, and we call it training. We have to be consistent with our dog. Why should we be less consistent with our fellows? If we make a brute vicious we can end its life, and determine the result of our folly. No such end is possible when our folly is multiplied in the consciousness of our fellow men.

Already we have caught a glimpse of the measure of memory.

It is never a negation; it is always a fact. It is never inaction; it is always action. It dominates motive, for good or for evil. It affects judgment. It builds the under structure of which the pinnacle is *I*.

There is no definition of memory, save that it is the influential record of a past activity. We have abandoned the idea of new creations of force. We have come to recognise the power of the sun, as a daily providence in nature; augmenting that which is, and changing the face of the world, as it has made its depths. Something is our daily gain, nothing is lost. All is active, or stored energy. The terms require explanation. They formulate something of the unknown. They express Newton's notion of ourselves as children gathering pebbles on the shore of the great ocean of eternity. But the pebbles are real, even if the ocean be unknown. The most true cast of scientific mind worships when it cannot know, and gives thanks for what it can grasp. There is no atheism in science. The reverence for the known increases the reverence for that which lies beyond scientific proof.

There is a familiar example of stored energy in the coal which we use. Coal is simply buried and compressed wood. With that wood is also buried the heat and light which gave it growth, at a time when the world had no written history. That is stored energy.

The housemaid who lights the fire, the stoker who tends the engine, raise that stored energy for present use, and when the immediate purpose is served, the energy passes again into the stage of latency, waiting a fresh call. When stored energy is used for an immediate purpose, we speak of it as dissipated. Distributed is, perhaps, a term more easily understood. The energy is spread abroad, to be regathered in new combination at a later date. None of it is lost.

But coal is reduced to ash. Memory is not. We do not lose the memory which we impart to an acquaintance, we rather benefit by it. That is one spark of the Deity in man which blesses him who gives as well as him who receives. And what is true of the individual is true of the race. Our boasted civilisation is but accumulated energy freely spent. Our progress is the testing and questioning of how much we have yet in store.

Follow me through one other question before we approach the investigation of memory as a physical fact. A fact as definite as electricity, if less determined than arithmetic.

You have had a grave illness, and your life has been in danger. You have been weeks or months, or even a year or two, before you felt yourself equal to cope with your fellows as formerly. The memory of your illness is graven deep upon those who by kinship or by kindness are your friends. But what of yourself? You have been told of it, and you feel that what you have been told is true. But it is a second-hand truth, very much second-hand in importance to your memory of a similar illness in a friend. You have lost little or nothing of what you previously knew. It is your recent *uptake* which is at fault. And why? The activity, which in health goes to write memory in physical characters, during disease goes to combat an enemy and to preserve the structures most needful to animal life. During illness we go back to our babyhood, take nourishment, and sleep. Our recovery depends upon nutrition, or feeding. Our memory "uptake" fails when our surplus nutrition is called upon to resist the ravages of disease.

As we pursue the subject, we must beware of looking upon memory as a mere machine. No human faculty is a mere machine, but underlying each there is a mechanism which, with all reverence, and with a full sense of our own finality, we may examine and study.

The careful and reverent work of certain great men has unveiled for us some of the machinery of memory. Their work rests upon the known, and reaches towards the unknown. They are Agnostics in the true sense of the word. Some prejudice attaches to the term from the frequency with which it is applied to matters of religious belief. Such an application is outside of our subject. But in observation and in science this *frame* of mind is the only sure foothold.

*(To be continued.)*

